# *ITPA* Polymorphisms Are Associated with Hematological Side Effects during Antiviral Therapy for Chronic HCV Infection

**DOI:** 10.1371/journal.pone.0139317

**Published:** 2015-10-06

**Authors:** Raoel Maan, Adriaan J. van der Meer, Willem Pieter Brouwer, Elisabeth P. C. Plompen, Milan J. Sonneveld, Robert Roomer, Annemiek A. van der Eijk, Zwier M. A. Groothuismink, Bettina E. Hansen, Bart J. Veldt, Harry L. A. Janssen, Andre Boonstra, Robert J. de Knegt

**Affiliations:** 1 Department of Gastroenterology and Hepatology, Erasmus MC University Medical Center Rotterdam, Rotterdam, the Netherlands; 2 Department of Viroscience, Erasmus MC University Medical Center Rotterdam, Rotterdam, the Netherlands; 3 Toronto Centre for Liver Disease, Toronto Western & General Hospital, University Health Network, Toronto, Canada; National Taiwan University Hospital, TAIWAN

## Abstract

**Background/Objective:**

Genetic polymorphisms in the inosine triphosphatase (*ITPA*) gene have been associated with the protection from early ribavirin(RBV)-induced hemolytic anemia among patients with chronic hepatitis C virus (HCV) infection. The aim of the present study was to investigate the association between the functional *ITPA* variants and hematological side effects during antiviral therapy with pegylated interferon (PegIFN) and RBV.

**Patients and Methods:**

This cohort study included all consecutive Caucasian patients treated for chronic HCV infection with PegIFN and RBV between 2000 and 2009 for whom a serum sample was available for genetic testing. The predicted inosine triphosphate pyrophosphatase (ITPase) activity was based on the genotypes of the SNPs rs1127354 and rs7270101. Decline in hemoglobin (Hb) during antiviral therapy, as well as dose reductions, blood transfusions and use of erythropoietin were assessed.

**Results:**

In total, 213 patients were included. The predicted ITPase activity was normal among 152 (71%) patients; 61 (29%) patients had ITPase deficiency. By multivariable linear regression, RBV dose in mg per kilogram (Beta 0.09, 95%CI 0.04–0.13, p<0.001) and normal ITPase activity (Beta 0.89, 95%CI 0.64–1.14, p<0.001) were associated with more Hb decline at week 4 of treatment. Patients with normal ITPase activity underwent more dose adjustments of RBV than patients with ITPase deficiency (19(13%) vs 1(2%),p = 0.014) and received erythropoietin more frequently (12 (8%) vs 0 (0%),p = 0.024).

**Conclusion:**

Genetic variants in the *ITPA* gene protected against RBV treatment-induced anemia among Caucasian patients with chronic HCV infection. Patients with normal ITPase activity underwent more dose reductions of RBV and received erythropoietin more frequently.

## Introduction

Currently, there is a changing paradigm in the treatment of chronic hepatitis C virus (HCV) infection. Although it is expected that the efficacy and safety of antiviral therapy improves considerably with the introduction of direct acting antivirals (DAAs), the high costs may limit the availability of these new drugs. Therefore, pegylated interferon (PegIFN) and ribavirin (RBV) containing regimens are likely to remain important treatment options in many countries around the world, also in high-income countries. Furthermore, some studies showed that the addition of RBV to DAAs could be beneficial in selected cases [1, 2]. Unfortunately, PegIFN and RBV are associated with many side effects, including cytopenias. These cytopenias occur frequently and are the most important reasons for dose reductions [3, 4]. As these dose reductions compromise treatment efficacy [5, 6], it is of great importance to select patients who are at greatest risk for these hematological side effects. These patients may benefit from strategies to optimise treatment adherence, such as early administration of supportive hematopoietic growth factors.

Recently, two genetic polymorphisms in the inosine triphosphatase (*ITPA*) gene on chromosome 20 were shown to be associated with protection against early RBV-induced hemolytic anemia during therapy with PegIFN and RBV [7]. The first polymorphism concerns a missense variant in exon 2 (rs1127354), the second concerns a splicing-altering single nucleotide polymorphism (SNP) in intron 2 (rs7270101). These two functional variants cause ITPase deficiency, subsequently preventing the depletion of erythrocyte adenosine triphosphate (ATP) and oxidative damage on the erythrocyte membrane [8]. Although ITPase deficiency is protective against anemia, it has been associated with a greater decline in platelet count during PegIFN and RBV therapy [9, 10]. It has been suggested that patients with normal ITPase activity have a higher chance to develop thrombocytosis in reaction to the decline in hemoglobin (Hb). Although the exact mechanisms for this reactive thrombocytosis have not been completely elucidated, the increased stimulation of megakaryocyte-erythoroid progenitor cells by erythropoietin (EPO) production is thought to be of major importance [11, 12].

The association of the *ITPA* variants with the occurrence of RBV-induced hemolytic anemia has been previously assessed [9, 10, 13, 14]. However, these studies were based on patients included in randomised controlled trials with strict inclusion criteria and dosing rules. It can be questioned whether these findings also apply for the general patient population treated with PegIFN and RBV in field practice, where dose reductions are less strictly applied. Therefore, the aim of this study was to investigate the relationships between functional *ITPA* variants and hematological side effects of PegIFN and RBV therapy in routine daily practice. Secondly, the relations between these genetic polymorphisms and the occurrence of PegIFN and RBV dose reductions, administration of EPO and blood transfusions and virological response to antiviral therapy was analysed.

## Patients and Methods

### Patients

All Caucasian patients of whom a blood sample was available for genetic testing were included from our previously described cohort, which includes all consecutive patients with chronic HCV infection who were treated with PegIFN alfa-2a or -2b and RBV between 2000 and 2009 in our center [15, 16]. The inclusion and exclusion criteria for this study are described elsewhere [15, 16]. Briefly, patients were included if they were treated with PegIFN and RBV between 2000 and 2009. Patients treated with conventional interferon and patients co-infected with human immunodeficiency virus or the hepatitis B virus were excluded. In order to prevent confounding by ethnic origin, only Caucasian patients were included in the present study.

The study was conducted in accordance with the guidelines of the Declaration of Helsinki and the principles of Good Clinical Practice. The ethical review board of the Erasmus Medical Center, Rotterdam, The Netherlands approved this study as it was considered to be a low-risk study using retrospective and anonymized patient data. Written informed consent was obtained from each patient for storage of serum samples.

### Data acquisition

We obtained baseline data on gender, age, race, body mass index, METAVIR score, HCV genotype, previous interferon-based treatment, platelet count, absolute neutrophil counts, Hb, bilirubin and albumin concentration, glucose levels, presence of hemophilia and use of anticoagulants and antiplatelet therapy, diabetes mellitus (DM), history of heroin use and/or smoking.

During therapy all patients visited the outpatient clinic in one to six weeks intervals. At every visit blood tests were performed and patients were assessed for dose reductions and discontinuation of antiviral therapy. Among patients who were treated within a standard of care protocol, PegIFN and RBV dose reductions were made at the discretion of the treating physician. Patient characteristics such as age, physical condition, virological response, comorbidities and side effects of antiviral therapy were taken into account when considering a dose reduction. All study protocols of the clinical trials stated that dose reductions should be made according to product labels. However, these guidelines were not applied in some patients, due to the expected decrease of antiviral efficacy. Therefore these patients were treated at the discretion of the treating physician as well. The use of blood transfusion and EPO were also registered.

### Endpoints

The primary endpoint was the decline in Hb (mmol/L) and platelet count (*10^9^/L) which was assessed at week 4 (+/-7 days). This time point was chosen in order to limit the influence of dose reductions on these hematological outcomes [7]. A clinically significant decline in Hb was defined as a decrease of at least 3.0 g/dL (1.86 mmol/L) or an absolute value lower than 10 g/dL (6.21 mmol/L). These thresholds were also used in other studies on ITPase deficiency [13, 14, 17]. Anemia was defined according to the thresholds used by the World Health Organization; for women a Hb concentration below 7.45 mmol/L and for men below 8.1 mmol/L were used as cut-off. Thrombocytopenia was defined as a platelet count below 150*10^9^/L. A clinical relevant thrombocytopenia was defined as a platelet count below 50*10^9^/L, since current guidelines advise to reduce the dose of PegIFN when platelet counts fall below 50*10^9^/L [18].

As a secondary endpoint, the decline in Hb and platelet counts were also assessed at week 8 and 12 (+/-7 days) of antiviral therapy, as well as the nadir values of these parameters.

Sustained virological response (SVR) was defined as HCV RNA negativity in blood six months after cessation of antiviral therapy. Dose reductions of RBV and PegIFN, as well as the administration of EPO and blood transfusion, were considered as clinical endpoints.

### Genotyping methods

Serum samples stored at -20° or -80° Celsius were used for DNA extraction and genotyping procedures, which were carried out centrally at LGC genomics. Purified genomic DNA of ≥5 ng was used for genotyping. Genotypes were assigned using all of the data from the study simultaneously. Genotype sequences were derived from NCBI. Genetic analyses were performed at the polymorphic sites rs12979860 (19:39248147, near *IL28B*, also known as interferon-ʎ3), rs1127354 (20:3213196, *ITPA*–1) and rs7270101 (20:3213247, *ITPA*–2). The *IL28B* SNP rs12979860 was chosen, since it best describes the association with sustained SVR for all genotypes [19–21]. Linkage disequilibrium and Hardy-Weinberg equilibrium (HWE) were tested for these SNPs using SNAP and OEGE [22].

### Predicted ITPase activity

As freshly acquired erythrocytes were lacking in order to directly measure ITPase activity, the predicted ITPase activity was based on genotypes of both *ITPA*–1 and *ITPA*–2 as is determined by previous analyses [23, 24]. Patients with normal ITPase activity (i.e. 100%) were defined as patients with the combined presence of CC-genotype and AA-genotype for rs1127354 and rs7270101 respectively (S1 Table). Patients with less than 100% ITPase activity were defined as patients with ITPase deficiency (non-CC-genotype and non-AA-genotype for rs1127354 and rs7270101 respectively, S1 Table).

### Statistical Analysis

Continuous variables were summarised as median (interquartile range [IQR]) and categorical variables as frequencies (percentages). Comparisons between groups were performed using X^2^ test for categorical variables or the Mann-Whitney U test for comparing medians. The genetic association analyses for *ITPA* and *IL28B* polymorphisms consisted of a dominant genetic model (CC- and AA-genotype vs non-CC- and non-AA-genotype for *ITPA* and CC-genotype vs non-CC genotype for *IL28B*) [14, 25]. Linear regression analysis determined which variables were associated with the absolute decline in Hb and platelet count at week 4. Logistic regression was performed to determine which variables were associated with a clinically significant Hb decline at week 4, SVR and virological relapse. For decline in Hb and platelet count, a sensitivity analysis was performed which excluded patients whom were treated with a high PegIFN dose induction regimen. Age, sex and variables with a p-value of ≤0.2 in univariable analyses were included in multivariable analyses. All final models were created by using a backward stepwise method, in order to select the variables that were significantly and independently associated. Potential confounding was checked. All statistical tests were two-tailed, and *p*<0.05 was considered to be statistically significant. The significance level of interactions was set at 0.01 in order to correct for multiple testing. SPSS version 21.0 (SPSS, Chicago, IL) was used.

## Results

### Patients

In total, 321 consecutive patients with chronic HCV infection were treated with PegIFN and RBV between 2000 and 2009, of which 256 were Caucasian. Two hundred thirteen (83%) of these patients, who had a sample available for genetic testing and could be genotyped for both *ITPA* polymorphisms, were included in the current analyses (S1 Fig). Median age was 45 years (IQR 39–50), 145 (68%) were male, 105 (49%) had HCV genotype 1, and 39 (18%) had cirrhosis. Of the included patients, 140 were treated within a standard of care protocol. The remaining 73 patients were treated within clinical studies: 61 patients participated in three clinical trials and received a standard of care with PegIFN alfa-2a (180μg/week) or -2b (1.5μg/kg/week) plus weight based RBV. The remaining 12 patients received a PegIFN induction regimen with either PegIFN alfa-2a (270–360 μg/week) for 24 weeks or PegIFN alfa-2b (2.0–3.0 μg/kg/week) for 24 weeks followed by 48 weeks of PegIFN and daily weight-based ribavirin.

### Genotyping

The majority of patients were homozygous carriers of the major allele for *ITPA*–1 (rs1127354 C) and *ITPA*–2 (rs7270101 A), respectively 200 (89%) and 170 (76%) patients. The minor allele frequency (MAF) was 0.04 for *ITPA*–1 (rs1127354 A) and 0.13 for *ITPA*–2 (rs7270101 C). Eighty-two of the patients (36%) had the favourable *IL28B* genotype (rs12979860 CC); MAF was 0.39 (rs12979860 T). All SNPs were in HWE and not in linkage disequilibrium (r^2^ ≤0.012). The call rates were 96% (217/226), 98% (221/226) and 96% (217/226), for *ITPA*–1, *ITPA*–2 and *IL28B* respectively.

S1 Table shows the distribution of the predicted ITPase activity according to the genotype of *ITPA*–1 and *ITPA*–2. In total, 152 (71%) patients had normal ITPase activity and 61 (29%) patients had ITPase deficiency. Baseline characteristics were compared between patients with normal ITPase activity and patients with ITPase deficiency (Table 1).

**Table 1:** Baseline characteristics.

Baseline variable	Total ^b^	Normal ITPaseactivity ^a^ ^,^ ^b^	ITPase deficiency ^a^ ^,^ ^b^	p-value
	N = 213	n = 152	n = 61	
Age	45 (39–50)	44 (38–49)	46 (40–54)	0.25
Male	145 (68%)	100 (66%)	45 (74%)	0.26
BMI in kg/m^2^ ^a^ ^,^ ^c^	26.0 (23.7–28.1)	25.8 (23.3–28.0)	26.4 (24.1–30.3)	0.13
HCV Genotype ^a^				0.20
1	105 (49%)	76 (50%)	29 (48%)	
2	19 (9%)	11 (7%)	8 (13%)	
3	76 (36%)	53 (35%)	23 (38%)	
4	13 (6%)	12 (8%)	1 (2%)	
Histology / elastography METAVIR score ^d^				0.47
F0-1	69 (32%)	54 (38%)	15 (28%)	
F2	67 (32%)	46 (33%)	21 (39%)	
F3	20 (9%)	16 (11%)	4 (7%)	
F4	39 (18%)	25 (18%)	14 (26%)	
Hemoglobin, in mmol/L	9.3 (8.7–9.9)	9.2 (8.7–9.9)	9.3 (8.7–9.9)	0.79
Anemia ^e^	8 (4%)	5 (3%)	3 (5%)	0.57
Platelet count, in platelet x 10^9^/L	197 (152–234)	198 (154–234)	191 (145–241)	0.38
Thrombocytopenia ^e^	48 (23%)	31 (20%)	17 (28%)	0.24
Absolute neutrophil count, in cells/μL ^c^	3200 (2500–4200)	3400 (2700–4400)	2800 (2300–3500)	**0.022**
Albumin, in g/L	44 (42–46)	44 (42–46)	44 (42–45)	0.22
Bilirubin, in μmol/L	10 (7–13)	10 (7–13)	10 (7–14)	0.75
Prothrombin time, in seconds	12.4 (11.7–13.2)	12.4 (11.7–13.2)	12.5 (11.8–13.5)	0.76
AST/ALT ratio ^a^ ^,^ ^c^	0.73 (0.54–1.0)	0.74 (0.55–0.98)	0.72 (0.52–1.0)	0.91
Gamma-gt, in IU/L ^c^	62 (33–118)	62 (33–111)	63 (39–150)	0.29
Creatinin, in mmol/L ^c^	71 (63–80)	71 (63–80)	70 (67–76)	0.57
HCV RNA load < 800,000 IU/mL ^a^ ^,^ ^c^	60 (28%)	35 (26%)	25 (46%)	**0.007**
Use of anticoagulants	7 (3%)	4 (3%)	3 (5%)	0.40
Presence of haemophilia	11 (5%)	8 (5%)	3 (5%)	0.92
Presence of DM ^a^	12 (6%)	11 (7%)	1 (2%)	0.11
Smoking	129 (61%)	92 (66%)	37 (69%)	0.71
History of IV drug use ^a^	121 (57%)	82 (55%)	39 (66%)	0.15
PegIFN alfa-2a ^a^	151 (71%)	112 (74%)	39 (64%)	0.16
PegIFN induction regimen ^a^	12 (6%)	10 (7%)	2 (3%)	0.36
Dose of RBV, in mg/kg	13.2 (11.8–14.5)	13.5 (12.0–14.6)	12.8 (10.3–14.1)	**0.036**

a. Abbreviations: ITPase, inosine triphosphaye pyrophosphatase; BMI, body mass index; HCV, hepatitis C virus; AST, aspartate aminotransferase; ALT, alanine aminotransferase; DM, diabetes mellitus; IV, intravenous; PegIFN, pegylated interferon; RBV, ribavirin

b. Medians are presented as number (IQR). Numbers are presented as n, (percentage of whole group)

c. Variables with a ‘c’ were missing in ≥ 10%

d. Liver biopsy or elastography was available in 195 patients

e. Anemia was defined as a Hb concentration below 8.1 mmol/L for men and below 7.45 mmol/L for women, thrombocytopenia was defined as a platelet count below 150*109/L

### ITPase deficiency and on-treatment hemoglobin concentration

**Fig 1:**
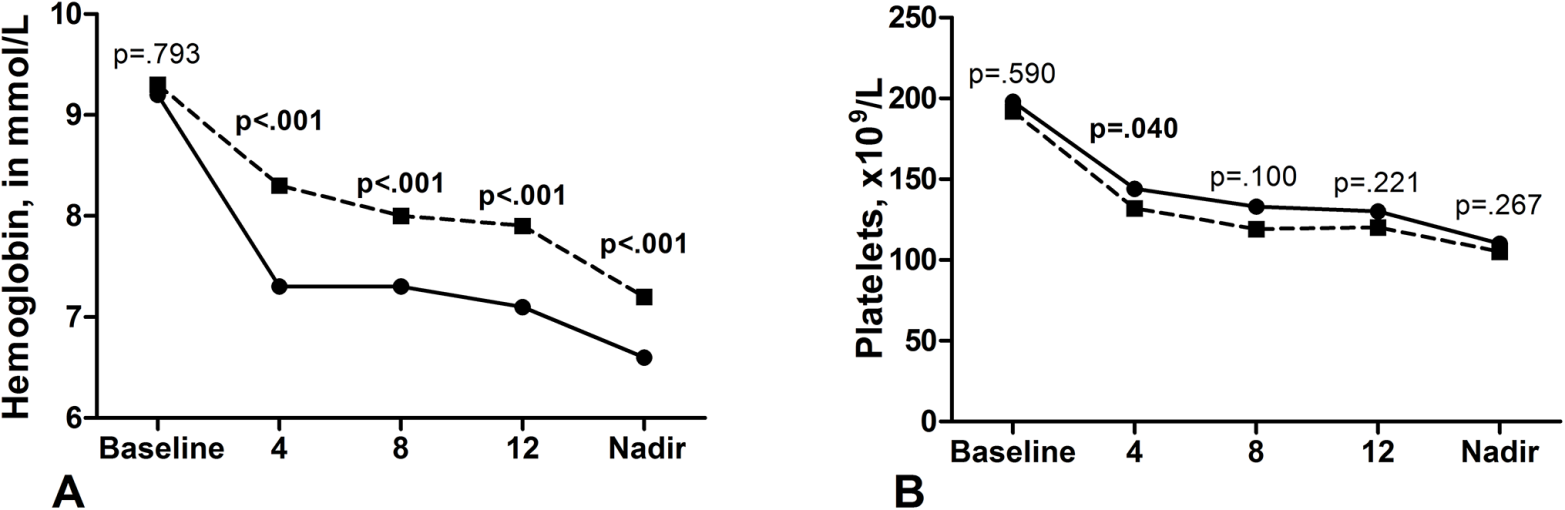
Median hemoglobin and platelet count. Median hemoglobin (A) and platelet counts (B) at baseline, at week 4, 8 and 12 and the nadir hemoglobin and platelet count during treatment. Dashed line represents the patients with ITPase deficiency and the black line represents patients with normal ITPase activity. Abbreviations: ITPase, inosine triphosphaye pyrophosphatase.

In total, 182 (85%) patients experienced at least one episode of anemia during antiviral treatment and in 157 (74%) patients a clinically significant decline in Hb was reported. At baseline, median Hb concentration was 9.2 mmol/L (IQR 8.7–9.9) for patients with normal ITPase activity and 9.3 mmol/L (IQR 8.7–9.9) for patients with ITPase deficiency (p = 0.793). At treatment weeks 4, 8 and 12, median Hb concentration was significantly lower among patients with normal ITPase activity (p<0.001 for all time points, Fig 1A). The nadir median Hb concentration was lower for patients with normal ITPase activity compared to patients with ITPase deficiency, respectively 6.6 mmol/L (IQR 5.8–7.2) and 7.2 mmol/L (IQR 6.6–8.1, p<0.001). Furthermore, patients with ITPase deficiency had a lower occurrence of a clinically significant decline in Hb as compared to patients without ITPase deficiency at week 4, 8 and 12 (p<0.001 for all timepoints, S2 Fig).

Multivariable linear regression analysis showed that baseline platelet count (Beta -0.02, 95% CI -0.04–-0.00, p = 0.022), baseline Hb concentration (Beta 0.44, 95% CI 0.29–0.59, p<0.001), RBV dose per kilogram body weight (Beta 0.09, 95% CI 0.04–0.13, p<0.001) and normal ITPase activity (Beta 0.89, 95% CI 0.64–1.14, p<0.001) were associated with the absolute decline in Hb concentration at week 4 of treatment (Table 2). The interaction terms between the variables in the final model were not statistically significant. Also in a sensitivity analysis for which patients with a PegIFN induction regimen were excluded the presence of normal ITPase activity was associated with the occurrence of a significant decline in Hb concentration at week 4 (Beta 0.91, 95% CI 0.65–1.18, p<0.001).

**Table 2:** Univariable and multivariable linear regression analysis for absolute hemoglobin decline at week 4.

Baseline variable	Univariable		Multivariable ^b^	
	Beta (95% CI) ^a^	p-value	Beta (95% CI) ^a^	p-value
Age, per year	0.05 (-0.09–0.02)	0.473	0.04 (-0.01–0.02)	0.535
Female gender	-0.09 (-0.38–0.20)	0.545	0.24 (-0.03–0.50)	0.086
Cirrhosis	0.09 (-0.26–0.44)	0.628		
DM ^a^	-0.01 (-0.60–0.59)	0.984		
BMI ^a^	-0.01 (-0.05–0.02)	0.490		
Platelet count, per 10x10^9^/L	-0.02 (-0.04–0.00)	0.115	-0.02 (-0.04–-0.00)	0.022
Hb, per mmol/L ^a^	0.36 (0.21–0.52)	<0.001	0.44 (0.29–0.59)	<0.001
PegIFN 2b vs PegIFN 2a	-0.08 (-0.38–0.22)	0.583		
PegIFN induction regimen ^a^	0.37 (-0.20–0.94)	0.203		
RBV dose, per mg/kg ^a^	0.11 (0.05–0.16)	<0.001	0.09 (0.04–0.13)	<0.001
Treatment naïve	0.05 (-0.31–0.42)	0.776		
Presence of hemophilia	0.44 (-0.21–1.09)	0.184		
Use of anticoagulants	-0.26 (-1.00–0.48)	0.490		
HCV Genotype (2/3 vs 1/4) ^a^	-0.38 (-0.65–-0.11)	0.006		
*IL28B* (CC vs CT/TT) ^a^	-0.12 (-0.40–0.17)	0.422		
*ITPA–1* (CC vs CA/AA) ^a^	1.02 (0.52–1.51)	<0.001		
*ITPA–2* (AA vs AC/CC) ^a^	0.81 (0.50–1.12)	<0.001		
Normal ITPase activity ^a^	0.93 (0.66–1.21)	<0.001	0.89 (0.64–1.14)	<0.001

a. Abbreviations: CI, confidence interval; DM, diabetes mellitus; BMI, body mass index; Hb, hemoglobin; PegIFN, pegylated interferon; RBV, ribavirin; HCV, hepatitis C virus; IL28B, interleukin-28B; ITPA, inosine triphosphatase; ITPase, inosine triphosphaye pyrophosphatase

b. The final model was created by using a backward stepwise method. Confounding was checked.

In multivariable logistic regression analysis, the occurrence of a significant decline, as a dichotomous variable, was associated with baseline Hb concentration (OR 2.31, 95% CI 1.47–3.64, p<0.001), RBV dose per kilogram body weight (OR 1.41, 95% CI 1.19–1.67, p<0.001) and normal ITPase activity (OR 11.5, 95% CI 4.24–31.1, p<0.001) (S2 Table). Again, in a sensitivity analysis among patients without a PegIFN induction regimen, the presence of normal ITPase activity was associated with the occurrence of a significant decline in Hb concentration at week 4 (OR 13.4, 95% CI 4.61–39.0, p<0.001).

### ITPase deficiency and on-treatment platelet counts

Median platelet count was only significantly higher at week 4 of treatment among patients with normal ITPase activity compared to patients with ITPase deficiency (144x10^9^/L, (IQR 103–196) vs 132x10^9^/L, (IQR 99–160); p = 0.040, Fig 1B). At least one episode of thrombocytopenia was present among 166 (78%) patients, of which 22 (10%) had a platelet count below 50x10^9^/L. Only ten (5%) patients experienced a platelet count below 50 x 10^9^/L at week 4 of treatment. The occurrence of a platelet count below 50 x 10^9^/L among patients with normal ITPase activity (4%) and patients with ITPase deficiency (7%) was comparable (p = 0.418). When the whole treatment period was taken into account, the occurrence of a platelet count below 50 x 10^9^/L was still similar between patients with normal ITPase activity and ITPase deficiency (11% vs 10%, respectively, p = 0.881).

In multivariable linear regression analysis, adjusted for Hb decline, baseline platelet count (per 10 x 10^9^/L, Beta 2.55, 95%CI 1.73–3.38, p<0.001) and cumulative dose of PegIFN (per 100 mcg, Beta 4.86, 95%CI 2.75–6.98, p<0.001) were associated with more decline in platelet count at week 4, wheras the presence of normal ITPase activity (Beta -18.5, 95%CI -29.7–-7.31, p = 0.001) was significantly associated with less decline in platelet count at week 4 (Table 3). When patients with a PegIFN induction regimen were excluded, presence of normal ITPase activity was still associated with less decline in platelet count (Beta -16.5, 95%CI -27.6–-5.33, p = 0.004).

**Table 3:** Univariable and multivariable linear regression analysis for absolute decline in platelet count at week 4.

Baseline variable	Univariable		Multivariable ^b^	
	Beta (95% CI) ^a^	p-value	Beta (95% CI) ^a^	p-value
Age, per year	-0.75 (-1.35–-0.15)	0.015	-0.44 (-9.46–2.32)	0.233
Female gender	7.13 (-5.25–19.5)	0.257	6.15 (-4.69–17.0)	0.265
Cirrhosis	-5.10 (-19.8–9.62)	0.495		
DM ^a^	-3.23 (-28.2–21.8)	0.799		
BMI ^a^	-0.22 (-1.71–1.28)	0.776		
Platelet count, per 10x10^9^/L	2.49 (1.60–3.37)	<0.001	2.55 (1.73–3.38)	<0.001
Hb, per mmol/L ^a^	-1.06 (-8.04–5.92)	0.765		
Hb decline, per mmol/L ^a^	-7.89 (-13.8–-1.93)	0.010	-3.57 (-9.46–2.32)	0.233
PegIFN 2b vs PegIFN 2a ^a^	-15.0 (-27.5–-2.43)	0.020		
PegIFN induction regimen ^a^	13.9 (-10.0–37.9)	0.253		
Cumulative dose of PegIFN, per 100 mcg ^a^	4.45 (2.12–6.78)	<0.001	4.86 (2.75–6.98)	<0.001
RBV dose, per mg/kg ^a^	-1.21 (-3.61–1.19)	0.320		
HCV Genotype (2/3 vs 1/4) ^a^	3.69 (-8.00–15.4)	0.534		
*IL28B* (CC vs CT/TT) ^a^	-5.76 (-17.9–6.40)	0.351		
*ITPA–1* (CC vs CA/AA) ^a^	-18.2 (-39.8–3.26)	0.096		
*ITPA–2* (AA vs AC/CC) ^a^	-9.54 (-23.4–4.33)	0.177		
Normal ITPase activity ^a^	-14.9 (-27.5–-2.19)	0.022	-18.5 (-29.7–-7.31)	0.001

a. Abbreviations: CI, confidence interval; DM, diabetes mellitus; BMI, body mass index; Hb, hemoglobin; PegIFN, pegylated interferon; RBV, ribavirin; HCV, hepatitis C virus; IL28B, interleukin-28B; ITPA, inosine triphosphatase; ITPase, inosine triphosphaye pyrophosphatase

b. The final model was created by using a backward stepwise method. Confounding was checked.

### ITPase deficiency, dose reductions, EPO and blood transfusions

**Fig 2:**
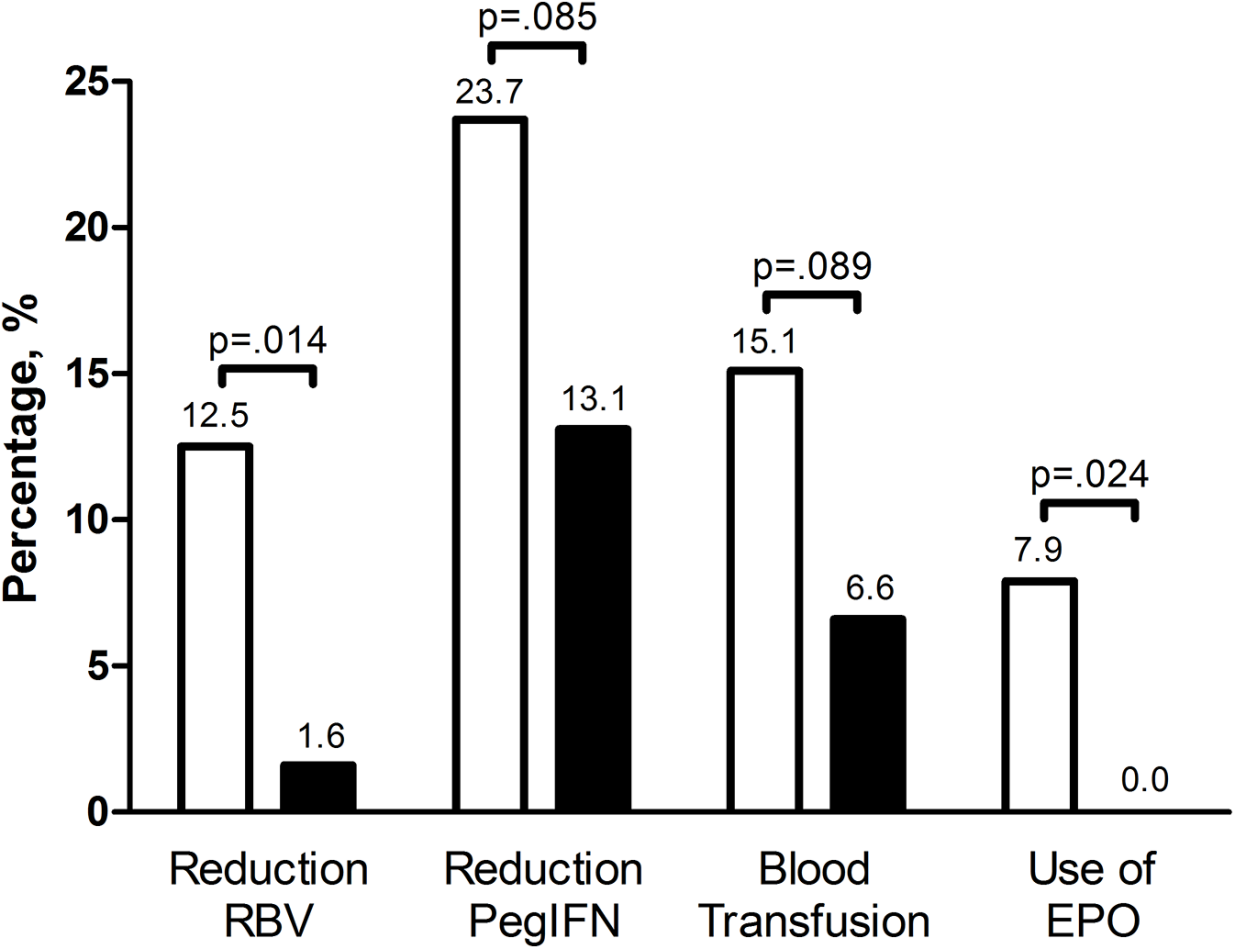
ITPase deficiency, dose reductions, EPO and blood transfusions. Percentage of patients with at least one dose reduction of RBV or PegIFN, at least one blood transfusion or one dose of EPO during treatment. White bars represent the patients with normal ITPase activity and the black bars represent patients with ITPase deficiency. Abbreviations: ITPase, inosine triphosphate pyrophosphatase; RBV, ribavirin; PegIFN, pegylated interferon; EPO, erythropoietin.

In total, 20 (9%) patients underwent at least one dose reduction of RBV and 44 (21%) patients at least one dose reduction of PegIFN. At least one blood transfusion was given to 27 (13%) patients and 12 (6%) patients received at least one dose of EPO. Nineteen (13%) patients with normal ITPase activity underwent at least one dose reduction of RBV, whereas one (2%) patient with ITPase deficiency underwent at least one dose reduction (p = 0.014, Fig 2). The dose of PegIFN was reduced among 36 (24%) patients with normal ITPase activity and among eight (13%) patients with ITPase deficiency (p = 0.085). Blood transfusion and EPO were administered to 23 (15%) and twelve (8%) patients with normal ITPase; and to 4 (7%) and none (0%) of the patients with ITPase deficiency (p = 0.089 and p = 0.024, respectively).

### ITPase deficiency and virologic response

In total, 123 (58%) patients attained SVR, 45 (21%) patients had a virological relapse and 43 (20%) were non-responder. Two patients, who were both HCV RNA negative at the end of treatment, were lost to follow-up before being able to assess the sustainability of their virological response. Neither polymorphisms in the *ITPA* gene (*IPTA–1* CC vs. CA/AA, OR 1.84 95% CI 0.66–5.15, p = 0.24; *ITPA–2* AA vs AC/CC, OR 1.03 95% CI 0.54–1.97, p = 0.92) nor the presence of normal ITPase activity (OR 1.23, 95% CI 0.68–2.24, p = 0.50) were associated with SVR. In multivariable logistic regression analyses, age (per year, OR 0.94, 95% CI 0.90–0.98, p = 0.004), baseline gamma-glutamyltransferase (per U/L, OR 0.99, 95% CI 0.99–1.00, p = 0.016), HCV genotype (2/3 vs 1/4, OR 4.26, 95% CI 1.89–9.63, p<0.001) and *IL28B* genotype (CC vs CT/TT, OR 5.06, 95% CI 2.16–11.9, p<0.001) were associated with SVR (S3 Table).

Univariable logistic regression analysis showed that the presence of normal ITPase activity was not significantly associated with virological relapse (OR 0.67, 95% CI 0.34–1.36, p = 0.27). Multivariable logistic regression analysis showed that only age (OR 1.05, 95% CI 1.01–1.10, p = 0.02) was significantly associated with virological relapse.

## Discussion

This large cohort study in a real world setting found that genetic polymorphisms in the *ITPA* gene, resulting in ITPase deficiency, were associated with less Hb decline during pegIFN and RBV therapy among Caucasian patients with chronic HCV infection. Patients with normal ITPase activity were at higher risk to undergo dose reductions of both PegIFN and RBV, receive blood transfusions and be administered EPO. These patients may benefit from early administration of supportive hematopoietic growth factors in order to improve treatment adherence. Furthermore it could be a helpful tool in the decision of adding RBV, which has shown to be of additional value for some IFN-free regimens [1, 2].

Patients with ITPase deficiency (27% of our cohort) experienced lower declines in Hb concentration compared to patients with normal ITPase activity during the first four weeks of antiviral treatment. As expected, besides the presence of normal ITPase activity, RBV dose was also associated with a significant decline in Hb at week 4. The results of the present study confirm observations described in previous studies [9, 13, 14]. More important, our study revealed that patients with normal ITPase activity underwent more dose adjustments for RBV (13% vs 2%), and more often EPO was administered (8% vs 0%). Two previous studies among patients with HCV genotype 2 and 3 found no association between the ITPase activity and the need for RBV dose reductions [9, 14]. In the first study, including 238 patients with HCV genotype 2 and 3, RBV dose was reduced in 35 patients (15%) [14]. The presence of ITPase deficiency was not statistically associated with a lower hazard ratio (HR) for RBV dose reductions (HR 0.80, 95% CI 0.35–1.71, p = 0.57). This study used weight-based RBV and the dose was not reduced until Hb concentration fell below 5.9 mmol/L (i.e. 9.5 g/dL). The second study included 349 patients with HCV genotype 2 and 3, which showed that 6% of the patients did not receive the full planned dose of RBV. This low rate can be attributed to the use of low-dose RBV (i.e. 800 mg/day) and the shorter treatment duration among this patients. In contrast, and in line with our data, a study among solely HCV genotype 1 infected patients did describe that the presence of ITPase deficiency resulted in fewer RBV dose reductions [13]. A high rate of RBV dose reductions was found in this study (47%), attributed to the fact that they included missed doses in this rate. Only four patients in this trial received EPO. In general, these data were based on clinical trial cohorts, solely including selected patients with specific HCV genotypes, different doses of RBV as well as limited use of blood transfusion and EPO. Furthermore these studies used strict dosing rules, instead of dose adjustments according to the treating physician, which is more representative for the clinical setting. Recently, another real-world study, also including all HCV genotypes, found that patients with ITPase deficiency required less RBV dose reductions and less EPO [17]. In 18% of the patients with ITPA deficiency a RBV dose reductions was required, whereas 41% of the patients with normal ITPase activity underwent at least one dose reductions of RBV. However, this study used strict dosing rules as well, which is not an optimal reflection of daily practice. Moreover, they did not report on the ethnic background of patients, which is essential in genetic studies [25].

The presence of ITPase deficiency was also associated with a deeper decline in platelet count at week 4 of treatment, which could be explained by the absence of thrombocytosis in reaction to the hemolytic anemia. This is in line with previous studies which showed that reduced ITPase activity, which protects against RBV-induced anemia, is associated with the occurrence of treatment-induced thrombocytopenia [9, 10, 26]. ITPase deficiency was not related to the occurrence of a platelet count below 50 x 10^9^/L, but this rarely happens among patients with chronic HCV infection who are treated with pegIFN and RBV. Indeed, also in the current study, only 10% of patients had a platelet count below 50 x 109/L during their treatment course. Nevertheless, it could be a predictive tool among patients with cirrhosis, who are more prone to develop severe thrombocytopenia [27].

We did not find ITPase deficiency to be associated with virological response to PegIFN and RBV, perhaps because of limited power in our current study. Data on the influence of ITPase activity on the virological response is inconclusive, probably due to the inclusion of various HCV genotypes, heterogeneity in treatment regimens and the various ways in which the association was analysed. Our results are in line with the largest study to date, among patients with HCV genotype 1, which did not show a relation between ITPase deficiency and SVR either [13]. Nevertheless, it could be hypothesised that the higher frequency of dose reductions among patients with normal ITPase activity would compromise treatment efficacy, as was reported previously [5, 6]. However, the mechanism by which anemia and ITPase deficiency influence virological response is still not fully unravelled.

The clinical importance of *ITPA* polymorphisms in the era of regimes with DAAs could be debated. Although limited data is available, previous studies among patients treated with triple therapy including telaprevir also showed that a *ITPA* polymorphism (rs1127354) was associated with the development of on-treatment anemia [28–30]. Unfortunately, these studies are solely among patients of Asian ancestry, and these patients are monoallellic for *ITPA* polymorphism rs7270101. In contrast to these results, the limited data available for Caucasian patients showed contrasting results[31]. Among patients with advanced hepatic fibrosis, *ITPA* polymorphisms were associated with the severity of Hb decline at week 4, but not at week 12 of therapy. This effect was attributed to the increased plasma levels of RBV after the first 4 weeks of triple therapy[32]. Recently, it was shown that *ITPA* polymorphisms were also associated with anemia during IFN-free therapy [33]. Studies on the impact of RBV dose reductions among patients treated with triple therapy, including PegIFN, RBV and boceprevir, demonstrated that a lower dose of RBV did not affect SVR rates [34, 35]. Moreover, RBV dose reductions among patients with HCV genotype 2 and 3, treated with IFN-free regimens, also suggested no effect on SVR rates [36, 37]. Nevertheless, as RBV will still be a component of IFN-free treatment regimens, ITPA polymorphisms can select patients who are prone to develop RBV-induced hemolytic anemia in order to apply more conservative and/ or earlier dose reductions or early administration of supportive agents. Secondly, it may be used as an additive tool to select a specific IFN-free regimen for the individual patient as not all regimens require addition of RBV for optimal virological efficacy. However, more data is needed in order to conclude on the clinical utility of these SNPs.

A limitation of this study is that RBV concentrations were not available. A previous study showed higher RBV concentrations at week 4 among patients with normal ITPase activity compared to patients with ITPase deficiency [9]. This could be explained by the reduced hemolytic anemia that was present in the patients with ITPase deficiency, generating a larger distribution volume for the intracellular forms of RBV, leading to lower extracellular concentrations of RBV. On the other hand, lower plasma concentrations of RBV could have led to the protection against anemia. In contrast, another study among 546 patients showed no association between ITPase deficiency and RBV levels (p = 0.11) [13]. Finally, due to the retrospective character of the study, we were not able to measure ITPase activity directly, as this requires freshly acquired erythrocytes. Nevertheless, classification of the predicted ITPase activity by combining the two genotypes has been suggested to be reliable [23, 24].

In conclusion, this real-world study showed that ITPase deficiency is associated with the protection against hemolytic anemia among Caucasian patients with chronic HCV infection who are treated with PegIFN and RBV. This led to less dose reductions of RBV and PegIFN as well as less administration of blood transfusions and EPO. Since treatment efficacy is hampered by dose reductions, patients with normal ITPase activity may benefit from early strategies in order to improve treatment adherence.

## Supporting Information

S1 Fig Study flow chart. Abbreviations: *ITPA*, inosine triphosphatase; SNP, single nucleotide polymorphism.
(TIF)

S2 Fig Clinically significant hemoglobin decline. Percentage of patients with a clinically significant decline in Hb within the first twelve weeks. A significant decline was defined as a decrease of at least 1.86 mmol/L (3.0 g/dL) or an absolute value lower than 6.21 mmol/L (10 g/dL). White bars represent the patients with normal ITPase activity and the black bars represent patients with ITPase deficiency. Abbreviations: Hb, hemoglobin; ITPase, inosine triphosphate pyrophosphatase.
(TIF)

S1 Table Predicted ITPase activity according to genotype of *ITPA*–1 and *ITPA*–2. (DOCX)

S2 Table Univariable and multivariable logistic regression analysis for significant Hb decline at week 4. (DOCX)

S3 Table Univariable and multivariable logistic regression analysis for SVR. (DOCX)

## Abbreviations

ITPA: inosine triphosphatase
HCV: hepatitis C virus
PegIFN: pegylated interferon
RBV: ribavirin
Hb: haemoglobin
EPO: erythropoietin
IQR: interquartile range
ITPase: inosine triphosphate pyrophosphatase
OR: odds ratio
CI: confidence interval
DAAs: direct acting antivirals
SNP: single nucleotide polymorphism
ATP: adenosine triphosphate
DM: diabetes mellitus
SVR: sustained virological response
DNA: deoxyribonucleic acid
NCBI: National Center for Biotechnology Information
IL28B: interleukin-28B
HWE: Hardy-Weinberg equilibrium
SNAP: SNP annotation and proxy search
OEGE: online encyclopedia for genetic epidemiology studies
MAF: minor allele frequency
RNA: Ribonucleic Acid
HR: hazard ratio

## References

[1] Afdhal N, Zeuzem S, Kwo P, Chojkier M, Gitlin N, Puoti M, et al Ledipasvir and Sofosbuvir for Untreated HCV Genotype 1 Infection. N Engl J Med. 2014 Epub 2014/04/15. 10.1056/NEJMoa1402454 . 24725239

[2] Ferenci P, Bernstein D, Lalezari J, Cohen D, Luo Y, Cooper C, et al ABT–450/r-Ombitasvir and Dasabuvir with or without Ribavirin for HCV. N Engl J Med. 2014 Epub 2014/05/06. 10.1056/NEJMoa1402338 . 24795200

[3] Fried MW. Side effects of therapy of hepatitis C and their management. Hepatology. 2002;36(5 Suppl 1):S237–44. Epub 2002/10/31. S0270913902001945 [pii] 10.1053/jhep.2002.36810 . 12407599

[4] Russo MW, Fried MW. Side effects of therapy for chronic hepatitis C. Gastroenterology. 2003;124(6):1711–9. Epub 2003/05/23. S0016508503003949 [pii]. . 12761728 10.1016/s0016-5085(03)00394-9

[5] McHutchison JG, Manns M, Patel K, Poynard T, Lindsay KL, Trepo C, et al Adherence to combination therapy enhances sustained response in genotype-1-infected patients with chronic hepatitis C. Gastroenterology. 2002;123(4):1061–9. Epub 2002/10/03. S0016508502002111 [pii]. . 12360468 10.1053/gast.2002.35950

[6] Shiffman ML, Ghany MG, Morgan TR, Wright EC, Everson GT, Lindsay KL, et al Impact of reducing peginterferon alfa-2a and ribavirin dose during retreatment in patients with chronic hepatitis C. Gastroenterology. 2007;132(1):103–12. Epub 2007/01/24. S0016-5085(06)02466-8 [pii] 10.1053/j.gastro.2006.11.011 . 17241864

[7] Fellay J, Thompson AJ, Ge D, Gumbs CE, Urban TJ, Shianna KV, et al ITPA gene variants protect against anaemia in patients treated for chronic hepatitis C. Nature. 2010;464(7287):405–8. Epub 2010/02/23. nature08825 [pii] 10.1038/nature08825 . 20173735

[8] Hitomi Y, Cirulli ET, Fellay J, McHutchison JG, Thompson AJ, Gumbs CE, et al Inosine triphosphate protects against ribavirin-induced adenosine triphosphate loss by adenylosuccinate synthase function. Gastroenterology. 2011;140(4):1314–21. Epub 2011/01/05. S0016-5085(10)01883-4 [pii] 10.1053/j.gastro.2010.12.038 . 21199653

[9] Rembeck K, Waldenstrom J, Hellstrand K, Nilsson S, Nystrom K, Martner A, et al Variants of the inosine triphosphate pyrophosphatase gene are associated with reduced relapse risk following treatment for HCV genotype 2/3. Hepatology. 2014;59(6):2131–9. Epub 2014/02/13. 10.1002/hep.27009 . 24519039

[10] Thompson AJ, Clark PJ, Singh A, Ge D, Fellay J, Zhu M, et al Genome-wide association study of interferon-related cytopenia in chronic hepatitis C patients. J Hepatol. 2012;56(2):313–9. Epub 2011/06/28. S0168-8278(11)00385-0 [pii] 10.1016/j.jhep.2011.04.021 21703177 PMC3634361

[11] Broudy VC, Lin NL, Kaushansky K. Thrombopoietin (c-mpl ligand) acts synergistically with erythropoietin, stem cell factor, and interleukin–11 to enhance murine megakaryocyte colony growth and increases megakaryocyte ploidy in vitro. Blood. 1995;85(7):1719–26. Epub 1995/04/01. . 7535585

[12] Cardier JE, Erickson-Miller CL, Murphy MJ Jr. Differential effect of erythropoietin and GM-CSF on megakaryocytopoiesis from primitive bone marrow cells in serum-free conditions. Stem Cells. 1997;15(4):286–90. Epub 1997/01/01. 10.1002/stem.150286 . 9253112

[13] Holmes JA, Roberts SK, Ali RJ, Dore GJ, Sievert W, McCaughan GW, et al ITPA genotype protects against anemia during peginterferon and ribavirin therapy but does not influence virological response. Hepatology. 2014;59(6):2152–60. Epub 2014/01/23. 10.1002/hep.27022 . 24449403

[14] Thompson AJ, Santoro R, Piazzolla V, Clark PJ, Naggie S, Tillmann HL, et al Inosine triphosphatase genetic variants are protective against anemia during antiviral therapy for HCV2/3 but do not decrease dose reductions of RBV or increase SVR. Hepatology. 2011;53(2):389–95. Epub 2011/01/29. 10.1002/hep.24068 . 21274861 PMC4892367

[15] Roomer R, Hansen BE, Janssen HL, de Knegt RJ. Thrombocytopenia and the risk of bleeding during treatment with peginterferon alfa and ribavirin for chronic hepatitis C. J Hepatol. 2010;53(3):455–9. Epub 2010/06/22. S0168-8278(10)00470-8 [pii] 10.1016/j.jhep.2010.04.013 . 20561709

[16] Roomer R, Hansen BE, Janssen HL, de Knegt RJ. Risk factors for infection during treatment with peginterferon alfa and ribavirin for chronic hepatitis C. Hepatology. 2010;52(4):1225–31. Epub 2010/09/11. 10.1002/hep.23842 . 20830784

[17] Clark PJ, Aghemo A, Degasperi E, Galmozzi E, Urban TJ, Vock DM, et al Inosine triphosphatase deficiency helps predict anaemia, anaemia management and response in chronic hepatitis C therapy. J Viral Hepat. 2013;20(12):858–66. Epub 2013/12/07. 10.1111/jvh.12113 . 24304455

[18] European Association for the Study of the L. EASL recommendations on treatment of hepatitis C 2014. J Hepatol. 2014;61(2):373–95. Epub 2014/05/14. S0168-8278(14)00309-2 [pii] 10.1016/j.jhep.2014.05.001 . 24818984

[19] Ge D, Fellay J, Thompson AJ, Simon JS, Shianna KV, Urban TJ, et al Genetic variation in IL28B predicts hepatitis C treatment-induced viral clearance. Nature. 2009;461(7262):399–401. Epub 2009/08/18. nature08309 [pii] 10.1038/nature08309 . 19684573

[20] Sarrazin C, Susser S, Doehring A, Lange CM, Muller T, Schlecker C, et al Importance of IL28B gene polymorphisms in hepatitis C virus genotype 2 and 3 infected patients. J Hepatol. 2011;54(3):415–21. Epub 2010/11/30. S0168-8278(10)00834-2 [pii] 10.1016/j.jhep.2010.07.041 . 21112657

[21] Suppiah V, Moldovan M, Ahlenstiel G, Berg T, Weltman M, Abate ML, et al IL28B is associated with response to chronic hepatitis C interferon-alpha and ribavirin therapy. Nat Genet. 2009;41(10):1100–4. Epub 2009/09/15. ng.447 [pii] 10.1038/ng.447 . 19749758

[22] Rodriguez S, Gaunt TR, Day IN. Hardy-Weinberg equilibrium testing of biological ascertainment for Mendelian randomization studies. Am J Epidemiol. 2009;169(4):505–14. Epub 2009/01/08. kwn359 [pii] 10.1093/aje/kwn359 19126586 PMC2640163

[23] Shipkova M, Lorenz K, Oellerich M, Wieland E, von Ahsen N. Measurement of erythrocyte inosine triphosphate pyrophosphohydrolase (ITPA) activity by HPLC and correlation of ITPA genotype-phenotype in a Caucasian population. Clin Chem. 2006;52(2):240–7. Epub 2005/12/31. clinchem.2005.059501 [pii] 10.1373/clinchem.2005.059501 . 16384889

[24] Sumi S, Marinaki AM, Arenas M, Fairbanks L, Shobowale-Bakre M, Rees DC, et al Genetic basis of inosine triphosphate pyrophosphohydrolase deficiency. Hum Genet. 2002;111(4–5):360–7. Epub 2002/10/18. 10.1007/s00439-002-0798-z . 12384777

[25] Lunetta KL. Genetic association studies. Circulation. 2008;118(1):96–101. Epub 2008/07/02. 10.1161/CIRCULATIONAHA.107.700401 118/1/96 [pii]. . 18591452

[26] Tanaka Y, Kurosaki M, Nishida N, Sugiyama M, Matsuura K, Sakamoto N, et al Genome-wide association study identified ITPA/DDRGK1 variants reflecting thrombocytopenia in pegylated interferon and ribavirin therapy for chronic hepatitis C. Hum Mol Genet. 2011;20(17):3507–16. Epub 2011/06/11. ddr249 [pii] 10.1093/hmg/ddr249 . 21659334

[27] Maan R, van der Meer AJ, Hansen BE, Feld JJ, Wedemeyer H, Dufour JF, et al Effect of thrombocytopenia on treatment tolerability and outcome in patients with chronic HCV infection and advanced hepatic fibrosis. J Hepatol. 2014;61(3):482–91. Epub 2014/05/02. S0168-8278(14)00284-0 [pii] 10.1016/j.jhep.2014.04.021 . 24780302

[28] Akamatsu S, Hayes CN, Tsuge M, Murakami E, Hiraga N, Abe H, et al Ribavirin dose reduction during telaprevir/ribavirin/peg-interferon therapy overcomes the effect of the ITPA gene polymorphism. J Viral Hepat. 2014 Epub 2014/06/17. 10.1111/jvh.12275 . 24930407

[29] Ogawa E, Furusyo N, Nakamuta M, Kajiwara E, Nomura H, Dohmen K, et al Clinical milestones for the prediction of severe anemia by chronic hepatitis C patients receiving telaprevir-based triple therapy. J Hepatol. 2013;59(4):667–74. Epub 2013/05/28. S0168-8278(13)00348-6 [pii] 10.1016/j.jhep.2013.05.017 . 23707372

[30] Suzuki F, Suzuki Y, Akuta N, Sezaki H, Hirakawa M, Kawamura Y, et al Influence of ITPA polymorphisms on decreases of hemoglobin during treatment with pegylated interferon, ribavirin, and telaprevir. Hepatology. 2011;53(2):415–21. Epub 2011/01/20. 10.1002/hep.24058 . 21246582

[31] Aghemo A, Grassi E, Rumi MG, D'Ambrosio R, Galmozzi E, Degasperi E, et al Limited utility of ITPA deficiency to predict early anemia in HCV patients with advanced fibrosis receiving Telaprevir. PLoS One. 2014;9(4):e95881 Epub 2014/04/25. 10.1371/journal.pone.0095881 PONE-D-13-48917 [pii]. 24760000 PMC3997406

[32] Boglione L, De Nicolo A, Cusato J, Cariti G, Di Perri G, D'Avolio A. Significant early higher ribavirin plasma concentrations in patients receiving a triple therapy with pegylated interferon, ribavirin and telaprevir. J Viral Hepat. 2014;21(4):260–3. Epub 2014/03/07. 10.1111/jvh.12170 . 24597694

[33] Asselah T ZS, Soriano V, Bronowicki JP, Lohse AW, Müllhaupt B, et al ITPA gene variants predict hemolytic ribavirin induced anaemia in patients treated with the interferon-free regimen of faldaprevir, BI 207127 and ribavirin in sound-C2. J Hepatol. 2013;58(Suppl. 1):S482.

[34] Poordad F, Lawitz E, Reddy KR, Afdhal NH, Hezode C, Zeuzem S, et al Effects of ribavirin dose reduction vs erythropoietin for boceprevir-related anemia in patients with chronic hepatitis C virus genotype 1 infection–-a randomized trial. Gastroenterology. 2013;145(5):1035–44 e5. Epub 2013/08/09. S0016-5085(13)01141-4 [pii] 10.1053/j.gastro.2013.07.051 . 23924660

[35] Sulkowski MS, Poordad F, Manns MP, Bronowicki JP, Rajender Reddy K, Harrison SA, et al Anemia during treatment with peginterferon Alfa-2b/ribavirin and boceprevir: Analysis from the serine protease inhibitor therapy 2 (SPRINT–2) trial. Hepatology. 2013;57(3):974–84. Epub 2012/10/20. 10.1002/hep.26096 . 23081753

[36] Jacobson IM, Gordon SC, Kowdley KV, Yoshida EM, Rodriguez-Torres M, Sulkowski MS, et al Sofosbuvir for hepatitis C genotype 2 or 3 in patients without treatment options. N Engl J Med. 2013;368(20):1867–77. Epub 2013/04/24. 10.1056/NEJMoa1214854 . 23607593

[37] Zeuzem S, Dusheiko GM, Salupere R, Mangia A, Flisiak R, Hyland RH, et al Sofosbuvir and Ribavirin in HCV Genotypes 2 and 3. N Engl J Med. 2014 Epub 2014/05/06. 10.1056/NEJMoa1316145 . 24795201

