# Supplementary figures and images for: ITPA Polymorphisms Are Associated with Hematological Side Effects during Antiviral Therapy for Chronic HCV Infection
Source: PLoS One. 2015 Oct 6;10(10):e0139317. doi: 10.1371/journal.pone.0139317 (PMC4595504; doi:10.1371/journal.pone.0139317)

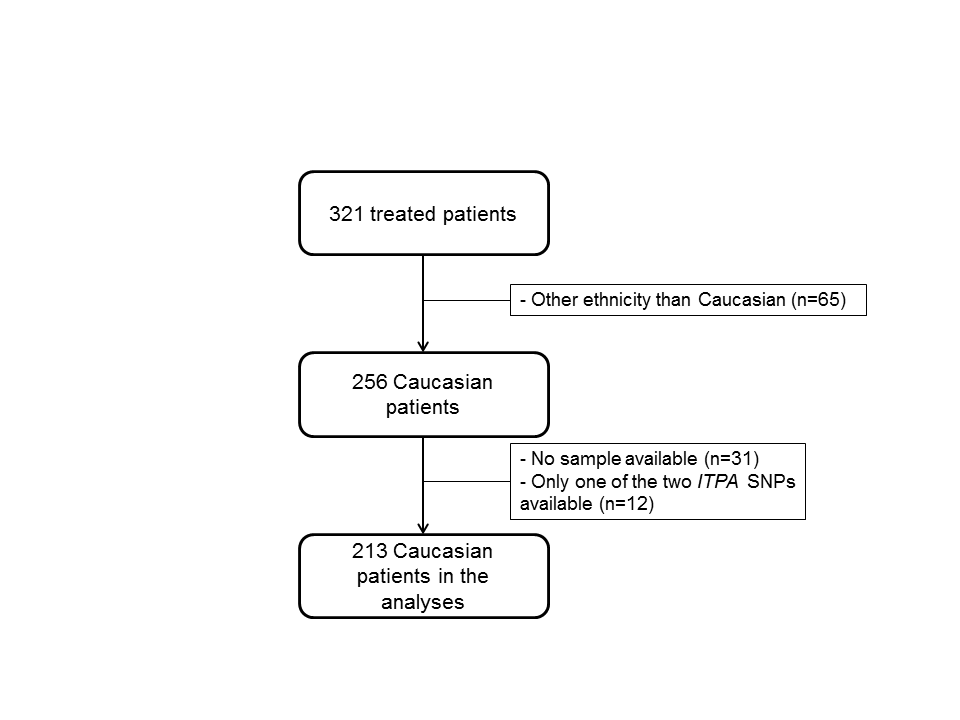

Supplement: S1 Fig — Abbreviations: ITPA, inosine triphosphatase; SNP, single nucleotide polymorphism. (TIF) [file pone.0139317.s001.tif]

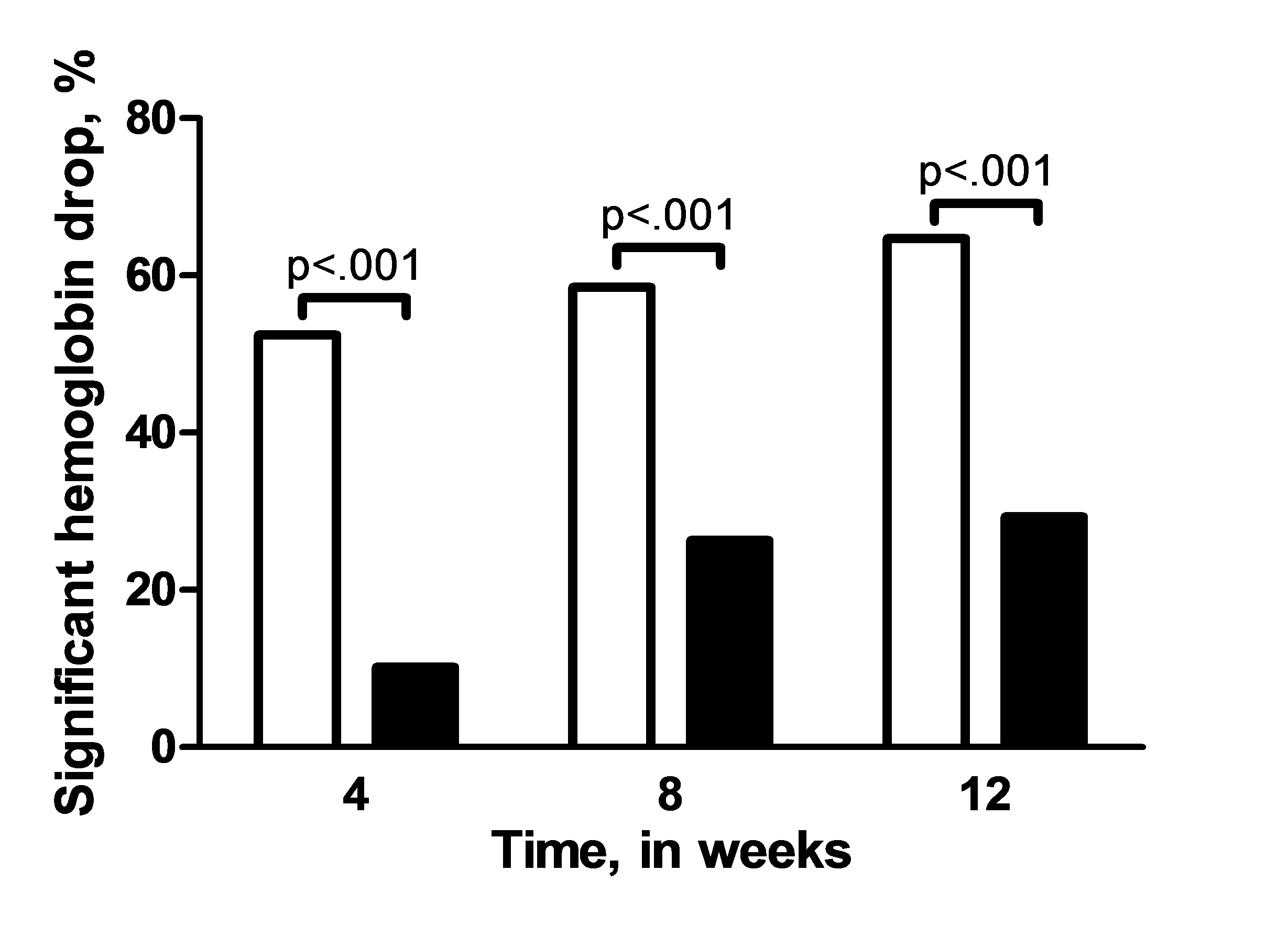

Supplement: S2 Fig — Percentage of patients with a clinically significant decline in Hb within the first twelve weeks. A significant decline was defined as a decrease of at least 1.86 mmol/L (3.0 g/dL) or an absolute value lower than 6.21 mmol/L (10 g/dL). White bars represent the patients with normal ITPase activity and the black bars represent patients with ITPase deficiency. Abbreviations: Hb, hemoglobin; ITPase, inosine triphosphate pyrophosphatase. (TIF) [file pone.0139317.s002.tif]
