# Supplementary material for: ITPA Polymorphisms Are Associated with Hematological Side Effects during Antiviral Therapy for Chronic HCV Infection
Source: PLoS One. 2015 Oct 6;10(10):e0139317. doi: 10.1371/journal.pone.0139317 (PMC4595504; doi:10.1371/journal.pone.0139317)
Supplement: S1 Table — (DOCX) [file pone.0139317.s003.docx]

**S1 Table. Predicted ITPase activity according to genotype of *ITPA*-1 and *ITPA*-2**

| ***ITPA*-1 genotype ^a^** | ***ITPA*-2 genotype ^a^** | **Predicted ITPase activity (%) ^a^** | **ITPase deficient ^a^** | **Distribution within cohort n (%)** |
| --- | --- | --- | --- | --- |
| Wild type (CC) | Wild type (AA) | 100 | No | 152 (67%) |
| Wild type (CC) | Heterozygote (AC) | 60 | Yes | 40 (18%) |
| Wild type (CC) | Homozygote (CC) | 30 | Yes | 5 (2.2%) |
| Heterozygote (CA) | Wild type (AA) | 25 | Yes | 12 (5.3%) |
| Heterozygote (CA) | Heterozygote (AC) | 10 | Yes | 4 (1.8%) |
| Homozygote (AA) | Wild type (AA) | <5 | Yes | 0 (0%) |

1. Abbreviations: ITPase, inosine triphosphate pyrophosphatase; ITPA, inosine triphosphatase
