# Supplementary material for: ITPA Polymorphisms Are Associated with Hematological Side Effects during Antiviral Therapy for Chronic HCV Infection
Source: PLoS One. 2015 Oct 6;10(10):e0139317. doi: 10.1371/journal.pone.0139317 (PMC4595504; doi:10.1371/journal.pone.0139317)
Supplement: S2 Table — (DOCX) [file pone.0139317.s004.docx]

**S2 Table. Univariable and multivariable logistic regression analysis for significant hemoglobin decline at week 4**

| **Baseline variable** | **Univariable** | | **Multivariable ^c^** | |
| --- | --- | --- | --- | --- |
|  | **OR (95% CI) ^a^** | **p-value** | **OR (95% CI) ^a^** | **p-value** |
| Age, per year | 1.01 (0.98-1.04) | 0.49 | 1.00 (0.96-1.04) | 0.95 |
| Female gender | 0.74 (0.40-1.36) | 0.33 | 1.07 (0.47-2.44) | 0.87 |
| Cirrhosis | 1.43 (0.70-2.90) | 0.33 |  |  |
| DM ^a^ | 1.06 (0.33-3.47) | 0.92 |  |  |
| BMI ^a^ | 0.98 (0.91-1.05) | 0.55 |  |  |
| Platelet count, per 10 x10^9^/L | 0.98 (0.93-1.02) | 0.31 |  |  |
| Hb, per mmol/L ^a^ | 1.69 (1.18-2.42) | 0.004 | 2.32 (1.48-3.65) | <0.001 |
| PegIFN 2a vs PegIFN 2b ^a^ | 1.29 (0.69-2.39) | 0.42 |  |  |
| PegIFN induction regimen ^a^ | 2.17 (0.67-7.10) | 0.198 |  |  |
| RBV dose, per mg/kg ^a^ | 1.31 (1.14-1.50) | <0.001 | 1.41 (1.19-1.67) | <0.001 |
| Treatment naïve | 0.58 (0.28-1.20) | 0.143 |  |  |
| Presence of hemophilia | 2.32 (0.63-8.48) | 0.204 |  |  |
| Use of anticoagulants | 0.58 (0.11-3.07) | 0.52 |  |  |
| HCV Genotype (2/3 vs 1/4) ^a^ | 0.36 (0.20-0.65) | 0.001 | 0.58 (0.27-1.22) | 0.15 |
| *IL28B* (CC vs CT/TT) ^a^ | 0.99 (0.55-1.76) | 0.96 |  |  |
| *ITPA-1* (CC vs CA/AA) ^a,^ ^b^ |  |  |  |  |
| *ITPA-2* (AA vs AC/CC) ^a^ | 6.42 (2.58-16.0) | <0.001 |  |  |
| Normal ITPase activity ^a^ | 9.72 (3.93-24.0) | <0.001 | 12.1 (4.48-32.8) | <0.001 |

1. Abbreviations: CI, confidence interval; DM, diabetes mellitus; BMI, body mass index; Hb, hemoglobin; PegIFN, pegylated interferon; RBV, ribavirin; HCV, hepatitis C virus; IL28B, interleukin-28B; ITPA, inosine triphosphatase; ITPase, inosine triphosphaye pyrophosphatase
2. Within the CA/AA genotype no significant decline in Hb occurred
3. The final model was created by using a backward stepwise method. Confounding was checked.
