# Supplementary material for: ITPA Polymorphisms Are Associated with Hematological Side Effects during Antiviral Therapy for Chronic HCV Infection
Source: PLoS One. 2015 Oct 6;10(10):e0139317. doi: 10.1371/journal.pone.0139317 (PMC4595504; doi:10.1371/journal.pone.0139317)
Supplement: S3 Table — (DOCX) [file pone.0139317.s005.docx]

**S3 Table. Univariable and multivariable logistic regression analysis for SVR**

| **Baseline variable** | **Univariable** | | **Multivariable ^b^** | |
| --- | --- | --- | --- | --- |
|  | **OR (95% CI) ^a^** | **p-value** | **OR (95% CI) ^a^** | **p-value** |
| Age, per year | 0.93 (0.90-0.96) | <0.001 | 0.93 (0.89-0.98) | 0.002 |
| Female gender | 1.85 (1.01-3.38) | 0.046 |  |  |
| Cirrhosis | 0.29 (0.14-0.61) | 0.001 | 0.38 (0.13-1.1) | 0.07 |
| DM ^a^ | 0.35 (0.10-1.18) | 0.090 | 0.54 (0.08-3.5) | 0.51 |
| BMI ^a^ | 0.95 (0.88-1.01) | 0.12 |  |  |
| Gamma-glutamyltransferase, per U/L | 0.99 (0.98-0.99) | <0.001 | 0.99 (0.99-1.00) | 0.004 |
| Baseline platelet count (per 10 x 10^9^/L) | 1.08 (1.03-1.14) | 0.002 |  |  |
| HCV Genotype 2/3 vs 1/4 ^a^ | 7.44 (3.91-14.2) | <0.001 | 4.55 (2.02-10.2) | <0.001 |
| PegIFN 2a vs PegIFN 2b ^a^ | 0.98 (0.54-1.79) | 0.95 |  |  |
| Treatment naïve | 1.78 (0.87-3.64) | 0.11 |  |  |
| HCV RNA < 800,000 IU/mL ^a^ | 1.55 (0.83-2.91) | 0.17 |  |  |
| *IL28B* (CC vs CT/TT) ^a^ | 4.04 (2.15-7.59) | <0.001 | 4.20 (1.85-9.55) | 0.001 |
| Significant Hb decline at week 4 ^a^ | 0.50 (0.28-0.88) | 0.017 |  |  |
| *ITPA*-1 CC vs CA/AA ^a^ | 1.84 (0.66-5.15) | 0.24 |  |  |
| *ITPA*-2 AA vs AC/CC ^a^ | 1.03 (0.54-1.97) | 0.92 |  |  |
| Normal ITPase activity ^a^ | 1.23 (0.68-2.24) | 0.50 |  |  |

1. Abbreviations: SVR, sustained virological response; CI, confidence interval; DM, diabetes mellitus; BMI, body mass index; HCV, hepatitis C virus; PegIFN, pegylated interferon; IL28B, interleukin-28B; Hb, hemoglobin; ITPA, inosine triphosphatase; ITPase, inosine triphosphaye pyrophosphatase
2. The final model was created by using a backward stepwise method. Confounding was checked.
